# Multidrug-Resistant TB in the Philippines: Totem and Taboo

**DOI:** 10.1371/journal.pmed.0030539

**Published:** 2006-12-26

**Authors:** Jose Luis Portero, Maria Rubio

Tupasi et al. [1] show the feasibility and cost-effectiveness of treating multidrug-resistant tuberculosis (MDR-TB) in a resource-limited setting. These conclusions could lead to error since the tertiary hospital where the pilot project was carried out is not representative of the average health-care setting available for the Filipino tuberculosis patient.

Resch et al. [2] highlight that this analysis is not an independent evaluation and that the intervention was set in ideal conditions. In the field, this pilot project will find serious difficulties in implementation within the government-run Filipino National Tuberculosis Program (NTP), where the majority of tuberculosis patients are diagnosed and treated. Moreover, in recent years Filipino tuberculosis health policy has not supported the development of capacities for mycobacteria culture and susceptibility tests in the public regional laboratories, missing the opportunity to strengthen competence on tuberculosis resistance management in the whole country. Taking into account the geography (an archipelago) and the structure of the NTP, a community-based pilot project carried out by the governmental health centers closer to the patients would have been more appropriate to study the feasibility and the real cost-effectiveness in a resource-limited setting.

The selection of 118 patients out of 171 eligible may bias the final results increasing the cure rate, since the most difficult patients to treat, by any circumstance, are also more problematic to enroll. A long-term follow-up of the patients may modify the final cure rate. It would have been important to analyze if any failure patient developed further resistances during the treatment, and the related public health implications, as well as a more in-depth discussion of the resistance patterns and its relationship with the tailored treatment and the final outcome. Identification of independent variables linked with favorable outcome are needed to design future strategies. On the other hand, the absence of data about the HIV status of the patients is remarkable, considering the ideal conditions of the study and the increasing number of cases in the Philippines.

Cost-effectiveness analysis may be holistic and may contemplate the MDR-TB control program with the rest of the activities of the NTP in order to evaluate the convenience and priority of each intervention. Unfortunately, the study does not support comprehensible strategies to control MDR-TB in the community. Patients, families, social activists, and health personnel related to tuberculosis control can offer important points of view. A larger consensus may avoid the switch of MDR-TB control from taboo to totem.
